# Effects of 1,25-dihydroxyvitamin D3 on pathological changes in rats with diabetic cardiomyopathy

**DOI:** 10.1186/s12944-017-0498-2

**Published:** 2017-06-08

**Authors:** Xiaoyun Zeng, Xintian Yu, Shan Xiao, Hua Yao, Jun Zhu

**Affiliations:** 1 Department of Endocrinology, the First Affiliated Hospital of Xinjiang Medical University, No. 137, Liyushan South Road, Urumqi, Xinjiang 830054 China; 2 Prevention, Diagnosis and Treatment Center of Diabetes, the First Affiliated Hospital of Xinjiang Medical University, Urumqi, Xinjiang 830054 China; 3 Xinjiang Key Laboratory of Metabolic Disease, the First Affiliated Hospital of Xinjiang Medical University, Urumqi, Xinjiang 830054 China

**Keywords:** 1,25-dihydroxyvitamin D3, Diabetic cardiomyopathy, Fas/FasL, Myocardial apoptosis

## Abstract

**Background:**

The role of 1,25-dihydroxyvitamin D3 (vitamin D) in the apoptosis of diabetic cardiomyopathy (DCM) is unclear. This study is to investigate the effects of vitamin D on the pathological changes in rats with DCM.

**Methods:**

Rats were randomly divided into the control, model, and treatment groups. DCM model was established by the high-fat and -sugar diet. Plasma glucose, body weight, heart weight, heart weight index, and serum levels of lactate dehydrogenase (LDH) and creatine kinase (CK) were determined. Heart tissue morphology was detected with histochemical staining. Expression levels of Fas and FasL were detected with RT-PCR and immunohistochemistry.

**Results:**

Compared with the control group, the body weights and heart weights were significantly declined, while the plasma glucose levels and heart weight indexes were significantly elevated, in the model group (*P* < 0.05). However, vitamin D significantly reversed the pathological changes in DCM rats (*P* < 0.05). Moreover, the serum levels of LDH and CK were significantly increased in the models, which were significantly decreased by vitamin D (*P* < 0.05). HE staining showed that, vitamin D significantly alleviated the histological changes of myocardial cells in DCM rats. In addition, the mRNA and protein expression levels of Fas and FasL were significantly elevated in the models (*P* < 0.05), which were significantly declined by vitamin D (*P* < 0.05).

**Conclusion:**

Vitamin D could alleviate pathological changes, reduce Fas/FasL expression, and attenuate myocardial cell apoptosis in DCM rats, which might be used as a potential effective therapy for the disease.

## Background

Diabetic cardiomyopathy (DCM) is a specific cardiac complication, which is chronic pathological change of cardiac muscle in response to the acute reactions caused by diabetes. These acute reactions mainly include abnormal gene expression, altered signal transduction, and cellular apoptosis [1]. Recent studies have shown that apoptosis of myocardial cells plays an important role in the pathogenesis and development of DCM [2, 3].

Fas receptor belongs to the tumor necrosis factor receptor family [4], which mainly exists in the form of membrane receptor. Fas plays an important role in the signal transduction in cellular apoptosis. It could bind to its natural ligand FasL, causing the cross-link and subsequent activation of Fas-associated death domain (FADD), further initiating apoptotic process [5]. Studies have shown that the elevated expression of Fas and FasL could enhance cellular apoptosis, while the declined expression could attenuate the apoptotic process [6, 7].

1,25-dihydroxyvitamin D3 is able to interact with the vitamin D receptor (VDR) and participates in the gene regulation during various biological processes, including the mineral homeostasis, bone metabolism, immune regulation, and cell proliferation and differentiation [8–11]. VDR has been shown to be expressed in myocardial cells [12]. However, there are few studies concerning the role of vitamin D in the apoptotic process in DCM. In this study, the effects of vitamin D on the pathological changes in rats with DCM were investigated. The mRNA expression levels of Fas and FasL in the heart tissues were detected. The protective effects of vitamin D on apoptosis of myocardial cells in DCM rats and the possible mechanisms were studied.

## Methods

### Animal grouping and modeling

Totally 34 male Sprague Dawley (SD) rats (specific pathogen free; 4–6 w, weighing 80 ± 7 g) were provided by the Xinjiang Medical University Experimental Animal Center (Urumqi, Xinjiang, China). All the animal experimental procedures were approved by the Animal Care and Use Committee of the Xinjiang Medical University. These rats were randomly divided into the following groups: (1) the control group (*n* = 8); (2) the model group (*n* = 12), which contained DCM rat model; and (3) the treatment group (*n* = 14), in which the DCM rats were subjected to the treatment with 1,25-dihydroxyvitamin D3 (vitamin D).

The rats in the control group were fed with a standard diet, while the rats in the model and treatment groups were fed with high-fat and -sugar diet (containing 10% refining lard, 20% sucrose, 2% cholesterol, and 68% normal diet). After 6 w, the rats in the model and treatment group were administrated with low dosage (30 mL/kg) of streptozotocin (STZ; Sigma, St. Louis, MO, USA) dissolved in pH 4.5 lemon acid buffer via intravenous injection. The control rats were injected with the citrate buffer instead. After another week, the fasting plasma glucose (FPG) and 2-h plasma glucose (2-h PG) levels were determined for the rats in the model and treatment groups. FPG ≥ 7.0 mmol/L and/or 2-h PG ≥ 11.1 mmol/L, together with the increased consumption of food and water, and increased urine production, indicated successful model establishment. Six weeks later, the rats in the treatment group were daily administrated with vitamin D (Shanghai Roche Pharmaceutical Ltd., Shanghai, China) dissolved in 0.05 mL peanut oil (Shandong Luhua Group Co., Ltd., Shandong, China; 0.03 μg/kg/day) by gavage for 6 weeks, while the rats in the control and model groups were administrated with the same amount of peanut oil alone. Finally, 30 out of 34 rats completed the model establishment and drug administration (2 rats in the model group and 1 rat in the treatment group died during the experiments, probably due to diabetic complications or infection).

### Body weight determination and biochemical tests

After 12-h fasting, the rat body weights were recorded. The tail blood sample was first obtained, and the FPG was determined with the plasma glucose meter. After anesthesia, the blood sample from the posterior vena cava was harvested, and the serum levels of lactate dehydrogenase (LDH) and creatine kinase (CK) were analyzed with an automatic biochemical analyzer (Abx Diagnostics, Montpellier, France).

### Heart weight measurement

After blood sampling, the whole rat heart was removed via thoracotomy. After washing with saline and drying with filter paper, the heart weight was measured by the electronic balance. After weighing, the heart tissue was cut into pieces. One part was fixed in 10% formaldehyde, which was left for the immunohistochemical detection; and the other part was stored at −70 °C for the following detection with RT-PCR.

### HE staining

HE staining was conducted as previously published [13]. The fixed heart tissue was dehydrated and embedded. The tissue was cut into 5-μm sections on a microtome (Leica, Nussloch, Germany). HE staining was then performed, and the morphology of the heart tissue was observed under a light microscope.

### Immunohistochemistry

Immunohistochemistry was performed according to a modified procedures previously published [14]. The fixed tissue was cut into 5-μm section series. After washing with PBS, the section was treated with 0.3% H_2_O_2_ to eliminate the endogenous peroxidase activity. After another washing step, the section was incubated with rabbit anti-rat anti-Fas antibody (ab82419; 1:50 dilution; Abcam, Cambridge, MA, USA) or rabbit anti-rat anti-FasL antibody (ab15285; 1:100 dilution; Abcam) at 4 °C overnight. Then horseradish peroxidase-conjugated goat anti-rabbit IgG (ZSGB-BIO, Beijing, China) was used to incubate the section at 37 °C for 30 min. After washing, color development was performed with DAB, following by contrast staining with hematoxylin, alcohol gradient dehydration, xylene clearing, and mounting with neutral gum.

Five fields were randomly selected from each slice under high magnification (400×), and the staining intensity was assessed as follows: 0, colorless; 1, pale yellow; 2, dark yellow; 3, brown. The percentage of positive cells was scored as follows: 0, all negative; 1, positive cells ≤25%; 2, 26%–50%; 3, 51%–75%; 4, > 75%. Staining intensity and positive cell percentage were added up to obtain an average score: 0, negative (−); 2–3, weak positive (+); 4–5, moderate positive (++); 6–7, strong positive (+++).

### Rt-Pcr

Total RNA was extracted with the Trizol agent (Invitrogen, Carlsbad, CA, USA). The cDNA was obtained with the RT-PCR kit (Fermentas, Maryland, NY, USA), according to the manufacturer’s instructions. The primers were synthesized by the Sangon, Shanghai, China: Fas, forward 5′-CTGTGGATCATGGCTGTCCTGCCT-3′ and reverse 5′-CTCCAGACTTTGTCCTTCATTTTC-3′; FasL, forward 5′-GGAATGGGAAGACACATATGGAACTGC-3′ and reverse 5′-CATATCTGGCCAGTAGTGCAGTAATTC-3′; and 18 s–RNA, reverse 5′-CGTTTATGGTCGGAACTACGA-3′. 20 μL PCR system consisted of 10 μL master mix, 2 μL cDNA, 0.2 μL forward and reversed primer each, and 7.6 μL ddH_2_O. PCR conditions for Fas and FasL were as follows: denaturation at 95 °C for 5 min; 94 °C for 60 s, 65 °C for 60 s, and 72 °C for 60 s, for 35 cycles; extension at 72 °C for 5 min. For 18 s–RNA, PCR conditions were as follows: denaturation at 95 °C for 5 min; 94 °C for 30 s, 60 °C for 60 s, 72 °C for 45 s, for 35 cycles; extension at 72 °C for 5 min. 5 μL PCR product was subjected to 1.5% agar agarose gel electrophoresis. The quantification of the target genes was performed with the Quantity One software.

### Statistical analysis

Data about continuous variables were expressed as mean ± SD. SPSS 17.0 software was used for statistical analysis. Student’s *t* test and one-way analysis of variance (ANOVA) were used for group comparison. Rank-sum test was used for the comparison of ranked data. *P* < 0.05 was considered statistically significant.

## Results

### Effects of vitamin D on physiological indexes in DCM rats

To investigate the effects of vitamin D on the physiological indexes in the DCM rat models, the plasma glucose levels, body weights, heart weights (heart weight indexes), and serum levels of LDH and CK were measured and compared for rats from the control, model, and treatment groups. Our results showed that, compared with the control group, the body weights and heart weights were significantly declined, while the plasma glucose levels and heart weight indexes were significantly elevated, in the model group (all *P* < 0.05). However, the body weights and heart weight were dramatically increased, while the plasma glucose levels were significantly decreased, by the treatment of vitamin D in the DCM rats (*P* < 0.05 for body weight and plasma glucose level) (Table 1). On the other hand, the serum levels of LDH and CK were significantly increased in the model group compared with the control group (*P* < 0.05), which were significantly decreased in the treatment group (*P* < 0.05) (Table 1). Taken together, these results suggest that, the treatment of vitamin D could significantly reverse the physiological alterations in the DCM rats.

**Table 1:** Effects of vitamin D on physiological indexes in DCM rats

Group	N	Plasma glucose level (mmol/L)	Body weight (g)	Heart weight (g)	Serum LDH level (U/L)	Serum CK level (IU/L)
Control	7	5.96 ± 0.90	481.00 ± 13.39	1.97 ± 0.11	143.43 ± 20.71	60.71 ± 14.64
Model	10	24.08 ± 2.99^*^	319.30 ± 38.43^*^	1.63 ± 0.21^*^	1664.70 ± 560.02^*^	719.10 ± 156.89^*^
Treatment	13	20.02 ± 2.43^*#^	363.54 ± 18.05^*#^	1.75 ± 0.22^*^	1086.08 ± 37.34^*#^	319.62 ± 71.89^*#^

Note: Compared with the control group, ^*^ *P* < 0.05; compared with the model group, ^#^ *P* < 0.05.

### Effects of vitamin D on myocardial cells in DCM rats

**Fig. 1:**
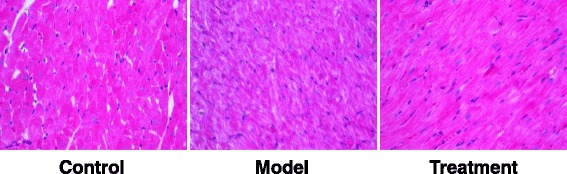
Histological detection of heart tissue in DCM rats. Histological characteristics of the heart tissues from the control, model, and treatment groups were detected with HE staining (400×)

To investigate the effects of vitamin D on the myocardial cells in these DCM rat models, the histological characteristics of these cells were detected with HE staining. As shown in Fig. 1, in the control group, the normal myocardial cells were neatly and tightly arranged, with clear structure and less extracellular matrix, and a small amount of fibroblasts were also observed. On the other hand, in the model group, hypertrophy and distortion were noted in the myocardial cells, which were irregularly arranged, with increased intercellular gap and interstitial and vascular extracellular matrix. However, in the treatment group, compared with the model group, the intercellular gap was dramatically reduced, and the interstitial and perivascular extracellular matrix was drastically decreased. These results suggest that, vitamin D treatment could significantly alleviate the histological changes in the myocardial cells in DCM rats.

### Effects of vitamin D on Fas and FasL expression in DCM rats

**Fig. 2:**
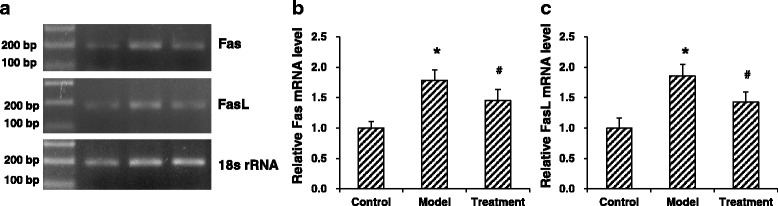
Effects of vitamin D on Fas/FasL mRNA expression levels in DCM rats. **a** The mRNA expression levels of Fas and FasL in the heart tissues from the control, model, and treatment groups were determined with RT-PCR. **b**-**c** Statistical analysis of the mRNA expression levels of Fas **b** and FasL **c**. Compared with the control group, ^*^ *P* < 0.05; compared with the model group, ^#^ *P* < 0.05

**Fig. 3:**
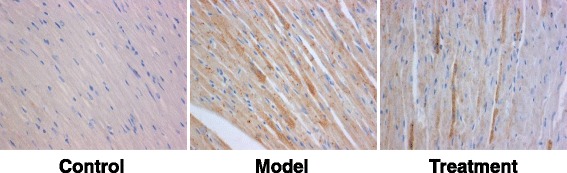
Effects of vitamin D on Fas/FasL protein expression levels in DCM rats. The protein expression levels of Fas and FasL in the heart tissues from the control, model, and treatment groups were detected with immunohistochemistry

To investigate the effects of vitamin D on the expression levels of Fas and FasL in the DCM rat models, RT-PCR and immunohistochemistry were performed to detect the mRNA and protein expression levels, respectively. Our results from RT-PCR showed that, compared with the control group, the mRNA expression levels of Fas and FasL were significantly elevated in the model group (*P* < 0.05). However, the treatment of vitamin D significantly declined the mRNA expression levels of Fas and FasL in the DCM rats (*P* < 0.05) (Fig. 2). Similar results were observed for the detection of Fas and FasL protein expression levels with immunohistochemistry. Our results showed that the protein expression levels of Fas and FasL were significantly increased in the model group compared with the control group (*P* < 0.05), which was significantly decreased in the treatment group (*P* < 0.05) (Fig. 3 and Tables 2 and 3). Taken together, these results suggest that, the mRNA and protein expression levels of Fas and FasL in the heart tissues are significantly elevated in the DCM rat models, which could be significantly declined by the treatment of vitamin D.

**Table 2:** Expression levels of Fas in the heart tissues in DCM rats

Group	Positive rate	Negative (−)	Weak positive (+)	Moderate positive (++)	Strong positive (+++)	χ^2^	*P*
Control	2 ± 0.34%	6	1	0	0	18.724	<0.01
Model	94 ± 6.17%	0	0	7	3		
Treatment	86 ± 9.28%	1	6	5	1

**Table 3:** Expression levels of FasL in the heart tissues in DCM rats

Group	Positive rate	Negative (−)	Weak positive (+)	Moderate positive (++)	Strong positive (+++)	χ^2^	*P*
Control	3 ± 0.96%	5	2	0	0	17.952	<0.01
Model	96 ± 7.45%	0	0	6	4		
Treatment	88 ± 10.31%	1	5	6	1		

## Discussion

Diabetic cardiomyopathy (DCM) is a major etiological factor inducing heart failure and ventricular diastolic dysfunction [15, 16]. In the present study, our results showed that, compared with normal control rats, several weeks of hyperglycemia lead to alterations of diabetic myocardial biochemical indicators (CK and LDH). In line with this, Zhang et al. [17] have shown that, serum levels of CK and LDH are significantly increased in streptozotocin-induced diabetic mouse models at 8 weeks. Moreover, Hou et al. [18] have also found that, streptozotocin and high-fat diet-induced diabetic cardiomyopathy are accompanied with significantly elevated CK-MB and LDH levels in rats at 16 weeks.

In this study, our results showed that the heart weights of diabetic rats were less than the normal control animals. This phenomenon may be due to the absence of effects of insulin on the heart cellular growth and protein synthesis. Consistent findings have been previously reported by Bugger et al. [19, 20]. Moreover, our results revealed that there were significant differences in myocardial cells between the diabetic and non-diabetic groups. Guo et al. [21] have shown that in the diabetic cardiomyopathy, the myocardium fibers are disordered, extensively collapsed, and degenerated. Similar results in the present study demonstrated the successful establishment of diabetic cardiomyopathy rat models. It was shown that, several weeks of hyperglycosemia would result in diabetic cardiomyopathy in rats. The results suggest that once diabetes is found, timely and effective control of hyperglycemia should be performed to prevent the occurrence and development of diabetic cardiomyopathy.

It has been shown that cardiomyocyte apoptosis is one of the crucial components in early cardiac responses, which may lead to devastating complications of cardiomyopathy [22–24]. Our results herein showed that the Fas/FasL system expression was up-regulated in the diabetic rat myocardial tissue, which regulated cellular apoptosis. The Fas pathway is critical for cardiomyocyte apoptosis, which could be easily activated by oxidative stress [25]. The Fas ligand, an integral membrane protein which binds to the Fas trimer, could induce conformational changes in Fas, thus enabling its cytoplasmic tail to recruit Fas-associated death domain protein (FADD) through interactions involving the death domains. Lin et al. [26] have reported that the Fas/Fas pathway-induced and caspase 8-mediated apoptosis is observed in the cardiomyocytes of STZ-treated DM rats. In line with these observations and the findings from Hegazy et al. [27], our results showed that, under poor control of diabetic plasma glucose, the expression of Fas and FasL was up-regulated in cardiomyocytes, which induced the expression of FADD pathway to increase the cellular apoptosis, representing one of the causes for diabetic cardiomyopathy.

At present, the effective treatments for DCM are still very limited. Recent studies have shown that, vitamin D could exert versatile effects on the cardiovascular system, which could play a role in the treatment of diabetes. In this study, our results showed that, compared with the model group, the plasma glucose level, heart weight, and myocardial enzyme levels were significantly reduced in the treatment group. Moreover, histochemical staining showed that the treatment of vitamin D effectively ameliorated the pathological changes in the DCM rats. These results suggest that vitamin D might be a potential efficient agent in treating diabetic cardiomyopathy [28].

Some studies have shown that vitamin D could exert anti-inflammatory, anti-oxidative, anti-proliferative, anti-fibrotic, and renin-angiotensin system-regulating effects in DCM [29, 30]. However, few studies have been focusing on the effects of vitamin D on apoptosis of myocardial cells, especially in DCM [29, 31]. Our results showed that, the mRNA and protein expression levels of Fas and FasL were significantly elevated in the DCM rats. However, the treatment of vitamin D could significantly down-regulate the expression levels of Fas and FasL in these model rats, at both the mRNA and protein levels. These results suggest that, vitamin D treatment could reduce the expression of Fas and FasL in the heart tissues, which might contribute to attenuate the apoptosis of myocardial cells in DCM.

## Conclusion

In conclusion, our findings demonstrated that vitamin D could be effective in protecting DCM. The protective effects of vitamin D in cardiac tissues might attribute to the down-regulated expression levels of Fas and FasL, indicating potential beneficial effects of apoptosis on diabetic cardiomyopathy. Vitamin D might be a promising potential therapeutic medicine to prevent diabetic cardiomyopathy.

## Abbreviations

2-h PG: 2-h plasma glucose
CK: Creatine kinase
DCM: Diabetic cardiomyopathy
FADD: Fas-associated death domain
FPG: Fasting plasma glucose
LDH: Lactate dehydrogenase
SD: Sprague Dawley
VDR: Vitamin D receptor
Vitamin D: 1,25-dihydroxyvitamin D3
